# Tumor Necrosis Factor (TNF) Bioactivity at the Site of an Acute Cell-Mediated Immune Response Is Preserved in Rheumatoid Arthritis Patients Responding to Anti-TNF Therapy

**DOI:** 10.3389/fimmu.2017.00932

**Published:** 2017-08-04

**Authors:** Rachel Byng-Maddick, Carolin T. Turner, Gabriele Pollara, Matthew Ellis, Naomi J. Guppy, Lucy C. K. Bell, Michael R. Ehrenstein, Mahdad Noursadeghi

**Affiliations:** ^1^Division of Infection and Immunity, University College London, London, United Kingdom; ^2^Division of Medicine, University College London, London, United Kingdom; ^3^Division of Neuropathology, Institute of Neurology, University College London, London, United Kingdom; ^4^UCL Advanced Diagnostics, London, United Kingdom; ^5^National Institute for Health Research, University College London Hospitals, Biomedical Research Centre, London, United Kingdom

**Keywords:** tumor necrosis factor, anti-tumor necrosis factor, rheumatoid arthritis, tuberculin skin test, transcriptional profiling

## Abstract

The impact of anti-tumor necrosis factor (TNF) therapies on inducible TNF-dependent activity in humans has never been evaluated *in vivo*. We aimed to test the hypothesis that patients responding to anti-TNF treatments exhibit attenuated TNF-dependent immune responses at the site of an immune challenge. We developed and validated four context-specific TNF-inducible transcriptional signatures to quantify TNF bioactivity in transcriptomic data. In anti-TNF treated rheumatoid arthritis (RA) patients, we measured the expression of these biosignatures in blood, and in skin biopsies from the site of tuberculin skin tests (TSTs) as a human experimental model of multivariate cell-mediated immune responses. In blood, anti-TNF therapies attenuated TNF bioactivity following *ex vivo* stimulation. However, at the site of the TST, TNF-inducible gene expression and genome-wide transcriptional changes associated with cell-mediated immune responses were comparable to that of RA patients receiving methotrexate only. These data demonstrate that anti-TNF agents in RA patients do not inhibit inducible TNF activity at the site of an acute inflammatory challenge *in vivo*, as modeled by the TST. We hypothesize instead that their therapeutic effects are limited to regulating TNF activity in chronic inflammation or by alternative non-canonical pathways.

## Introduction

Tumor necrosis factor (TNF) is a pleiotropic cytokine that is transcriptionally activated in response to a variety of stimuli during inflammation, infection, and stress. Its non-redundant role in the immune system is apparent in TNF-deficient mice, which have no phenotypic abnormalities in the steady state, but are unable to mount organized immune responses and thus suffer from an increased susceptibility to infection (1–4). Upon cellular activation, TNF is mostly produced by mononuclear phagocytes but is also expressed by neutrophils, lymphocytes, endothelial cells, and fibroblasts (5, 6). The membrane-bound precursor and the cleaved, soluble form of TNF are both biologically active and exert their effects *via* two structurally distinct transmembrane glycoprotein receptors. TNF receptor (TNFR) 1 is expressed on almost all nucleated cells and TNFR2 (p75) expression is restricted to cells of the immune system (7). Engagement of these receptors initiates intracellular signaling pathways leading to transcription of TNF-responsive genes, which in turn regulate cell proliferation and apoptosis or induce pro-inflammatory mediators (5, 8, 9).

Excessive TNF activity contributes to the complex pathogenesis of rheumatoid arthritis (RA) (10), associated with a pro-inflammatory cascade that includes the production of IL-1 and IL-6, and drives tissue destruction (11). The use of anti-TNF therapies in RA has substantially improved the outcome and clinical course of the disease (12). The five licensed TNF inhibitors comprise the anti-TNF antibodies infliximab, adalimumab, and golimumab, the TNFR2 Fc fusion protein etanercept (ETN), and the pegylated Fab fragment certolizumab. All of these were developed to competitively inhibit the binding of TNF to its cognate cellular receptors and consequently block its biological activity. However, a comprehensive characterization of their *in vivo* inhibitory effect on TNF activity has yet to emerge. Variable effects on the level of TNF itself in serum or synovium of RA patients have been described, which do not necessarily correlate with the clinical response to anti-TNF therapy (13–16). In general, the level of pro-inflammatory mediators in serum and synovium, and pro-inflammatory cytokine production by peripheral blood mononuclear cells (PBMC) is reduced following anti-TNF therapy (17–19). While these data suggest that anti-TNF therapies ameliorate the immunopathogenesis of RA, they do not reveal the contexts in which anti-TNF therapies regulate TNF activity. We do not know if they block inducible TNF activity in both acute and chronically inflamed tissues or if they mediate their effects by blocking TNF in the circulation or hematopoetic compartments, where TNF may have important roles in shaping systemic immune responses. These gaps in our knowledge limit further refinement of biological therapies for inflammatory diseases. Moreover, we have described at least one indirect mechanism of action, in which anti-TNF antibodies unexpectedly promoted an interaction between membrane-bound TNF on monocytes and TNFR2 on regulatory T cells leading to enhanced Treg activity that may contribute to disease control (20, 21). ETN also binds and neutralizes lymphotoxin α (LTA) (22), suggesting another putative mechanism for non-canonical effects of anti-TNF agents.

A well-recognized complication of anti-TNF therapy is increased susceptibility to granulomatous infections, especially with *Mycobacterium tuberculosis* (Mtb) (23, 24), in which cell-mediated immune responses are thought to represent the principal mechanism of host defense (25). The role of TNF in immune protection against tuberculosis was primarily derived from observations in TNFR deficient mice, which do not assemble well-formed granuloma (26, 27). This observation was replicated by administration of anti-TNF agents in wild type mice (28, 29). Consequently, increased risk of tuberculosis associated with anti-TNF therapy is also widely interpreted to be due to deficient TNF activity in cell-mediated immune protection, but direct evidence for this is lacking. Interestingly, anti-TNF antibodies such as infliximab and adalimumab invoke significantly greater risk of active tuberculosis in man, than the soluble TNFR, ETN (30–32). Possible mechanisms for the differential risk is reported to be apoptosis of monocytes and activated T cells (33–35), or depletion of Mtb reactive CD8 T cells by antibody binding to membrane TNF (36).

We have previously described transcriptional profiling at the site of the tuberculin skin test (TST) to make molecular and systems level assessments of *in vivo* human immune responses at the site of a standardized experimental challenge (37, 38). Clinical inflammation in the TST has been widely used as a surrogate for T cell memory for mycobacterial antigens (39), but transcriptional profiling of biopsies from the injection site reflects all the components of integrated innate and adaptive immune responses, each of which can be quantified with independently derived transcriptional modules (38, 40). Importantly, this approach also revealed immune responses in the absence of clinically evident inflammatory induration, allowing unprecedented sensitivity to measure immune responses that were previously described as anergic (37, 38). In the present study, we aimed to test the hypothesis that anti-TNF treated RA patients will exhibit attenuated TNF-dependent transcriptional responses at the site of the TST, and consequently evaluate the role of TNF in genome-wide assessments of cell-mediated immune responses.

## Materials and Methods

### Study Approval

This study was approved by UK National Research Ethics Service (reference no: 11/LO/1863).

### Study Population and Sampling

Healthy volunteers and adult patients with RA, treated with methotrexate (MTX), adalimumab, infliximab, or ETN, were invited to participate subject to selected criteria (Table 1). Written informed consent was obtained from all participants. Disease activity in RA patients was assessed using the Disease Activity Score of 28 commonly involved joints in RA (DAS28), comprising the number of swollen and tender joints, the erythrocyte sedimentation rate, and a visual analog scale of the patient’s subjective perception of pain marked out of 100 (41, 42).

**Table 1:** Inclusion and exclusion criteria for study participants.

Inclusion criteria	Exclusion criteria
Age 18–75 Male or female Confirmed diagnosis of rheumatoid arthritis (RA), treated with either adalimumab, infliximab, etanercept or methotrexate, with low disease activity Previous exposure to TB (previous active disease, previous positive tuberculin skin test, known contact with TB or from high demographic risk area)	Age <18 or >75 Other diseases of the immune system Other treatments affecting the immune system Very high disease activity of RA (DAS28 > 5.1) Previous history of reaction to lignocaine, purified protein derivative, or keloid scarring

Participants with positive peripheral blood IFNγ release assays as evidence of antimycobacterial T cell memory (see below) received either intradermal TST or saline injections in the volar aspect of the forearm as previously described (37, 38). At 72 h, the maximum diameter of inflammatory induration was measured, and two 3 mm adjacent punch biopsies were obtained over the center of the injection sites. In addition, whole blood was taken from further participants in each study group for *ex vivo* stimulation with 10 ng/mL recombinant human TNF (Life Technologies). Demographic, clinical, and laboratory data for each study group are summarized in Table 2 and for each participant in Data Sheet S3 in Supplementary Material, including all available data on previous blood IFNγ release assays or TB treatment. No participants had any evidence of active TB or received any treatment for TB during their participation in the study.

**Table 2:** Summary data for study participants undergoing skin tests.

Participant characteristics		Tuberculin skin test recipients				Saline recipients			
		HV	MTX	Mab	ETN	HV	MTX	Mab	ETN
Number		10	10	10	8	3	3	3	3

Age (median/range)		32.5 (28–42)	61 (53–69)	59.5 (38–75)	62.5 (30–67)	31 (29–38)	76 (57–76)	68 (66–74)%	36 (31–61)

Gender (% female)		60	80	60	62.5	10	100	66.7	100

Ethnicity	White British (%)	70	100	70	87.5	100	66.7	100	100
	White other (%)	20	0	10	0	0	0	0	0
	Asian Indian (%)	10	0	0	0	0	33.3	0	0
	Other (%)	0	0	20	12.5	0	0	0	0

RF status	Positive (%)	n/a	60	40	37.5	n/a	66.7	66.7	33.3
	Negative (%)	n/a	40	40	25	n/a	33.3	33.3	33.3
	Unknown (%)	n/a	0	20	37.5	n/a	0	0	33.3

CRP mg/dL (median/range)		n/a	2.3 (<0.6–11.0)	1.0 (<0.6–2.4)	3.2 (<0.6–11)	n/a	2.4 (2.1–2.5)	2.15 (<0.6–3.1)	0.9 (<0.6–0.9)

DAS28 (median/range)		n/a	2.0 (0.6–2.8)	2.6 (0.8–3.5)	2.3 (1.5–4.3)	n/a	3.0 (2.9–3.0)	3.6 (1.9–4.2)	2.4 (2.3–2.6)

Anti-CCP antibodies (%)		n/a	40	50	25	n/a	33	33	33

IFNγ ELISpots/2 × 10^5^ PBMC (median/range)		52.3 (11–00)	48.3 (10–25)	130.4 (15–300)	35.5 (10.5–38)	41 (15–98)	13.5 (13–50)	43.5 (38–60)	100 (20–122)

*HV, healthy volunteer; MTX, methotrexate only group; Mab, monoclonal antibody group; ETN, etanercept group; CRP, C-reactive protein; DAS28, disease activity score of 28 joints; CCP, cyclic citrullinated peptide; PBMC, peripheral blood mononuclear cells*.

### Peripheral Blood IFNγ Release Assays

Participants were screened for antimycobacterial T cell memory by peripheral blood IFNγ release assays using QuantiFERON-TB Gold tests (Qiagen) according to the manufacturer’s instructions or IFNγ ELISpot responses to purified protein derivative (PPD, Statens Serum Institute). Briefly, PBMC were obtained by density gradient centrifugation of heparinized whole blood with Ficoll-Paque PLUS (GE Healthcare Biosciences). Sterile, clear 96-well filter plate with a PVDF base membrane (Merck Millipore) were coated with 1 µg/mL antihuman IFNγ antibody (eBioscience) for 24 h at 4°C, prior to the addition of 2 × 10^5^ PBMC per well and PPD 10 µg/mL (SSI, Denmark), soluble anti-CD3 (HIT 3a) (eBioscience) as a positive control, or vehicle control. Plates were incubated for 48 h at 37°C. IFNγ production was detected using 1 µg/mL of biotinylated detection antibody (eBioscience), 1 µg/mL of streptavidin alkaline phosphatase (ALP) conjugate (Calbiochem) and BCIP/NBT substrate for ALP (Merck). The reaction was allowed to develop in the dark and stopped by addition of distilled water. Reactive foci were counted using AID ELISpot Software version 5.0 on the AID ELISpot Reader.

### Whole Genome Transcriptional Profiling and Analysis

Total RNA from peripheral blood, monocyte-derived macrophages (MDM), and skin biopsy samples was purified for genome-wide transcriptional profiling as previously described (37, 38). All novel gene expression data are available on ArrayExpress^1^ under accession numbers E-MTAB-5095 [lipopolysaccharide (LPS)/ETN-MDM module], E-MTAB-5094 (whole blood stimulation), and E-MTAB-5093 (skin biopsies). Differential gene expression was assessed in MultiExperiment Viewer v4.9.0.^2^ Two-tailed *t*-tests were used to derive TNF modules from *ex vivo* and cell culture experiments. Mann–Whitney tests were used to compare TST and saline injection sites. A *p* value of <0.05 was considered significant. This statistical threshold to identify significant differentially expressed genes was complemented by using a twofold change filter and statistical enrichment in upstream regulator analysis, performed using Ingenuity Pathway Analysis (Qiagen); and pathway analysis using InnateDB,^3^ visualized as network diagram in Gephi v0.8.2 beta.^4^ Principal component analysis was performed using the prcomp function in R.^5^

### Derivation of Transcriptional Modules

Derivation and validation of the TNF-MDM module was previously described (38). To generate the LPS/ETN-MDM module, human blood MDM cultures (43) were primed for 1 h ± 10 μg/mL ETN (Pfizer) before ultra-pure LPS (100 ng/mL; Invivogen) stimulation for 24 h (*n* = 3 per group). Transcriptional profiling of stimulated MDM was then performed as previously reported (38). LPS-inducible genes (>2-fold) were defined by comparison with transcriptional data from unstimulated MDM (*n* = 17). Among these LPS response genes, those that were attenuated significantly (>2-fold) in the presence of ETN were used as the LPS/ETN-MDM module. To derive a TNF-specific module in keratinocytes (TNF-KC module), we made use of previously published transcriptomic data from primary human keratinocytes (KCs) stimulated with a selection of cytokines, including TNF (44). Significant transcriptional responses (>4-fold) to 24 h TNF stimulation (10 ng/mL) were identified by comparison to unstimulated KCs, excluding genes that were also upregulated (>2-fold) by IFNγ, IL17 or IL22. The TNF-blood module was obtained by comparison of transcriptomes from whole blood (*n* = 4 healthy volunteers) incubated at 37°C for 3 h ±10 ng/mL recombinant human TNF, to identify transcripts that were significantly upregulated by >2-fold. The gene lists that make up each TNF module are listed in Data Sheet S1 in Supplementary Material. A human T cell-specific transcriptional signature (module M19) (45) and gene lists representing IFNγ activity and responses to Mtb (38, 40) have been published previously. Module scores represent the geometric mean log_2_ expression of all contributing genes and module enrichment represents the difference in module scores between data from TST and saline injection sites.

### Histology and Immunohistochemistry of Skin Biopsy Specimens

Punch skin biopsies for histological analysis were snap frozen in OCT Compound (Tissue-Tek). Frozen sections were carefully thawed and fixed in 4% neutral buffered formalin, then embedded in paraffin wax (Sakura). 3 µM sections were cut and stained using the Leica Bond III automated immunostaining platform, with the Leica Bond Polymer Refine detection kit (Leica DS9800) and a DAB chromogen. More specifically, antihuman antibodies against CD3 (clone LN10) (Leica NCL-L-CD3-565) and matrix metalloproteinase (MMP) 9 (clone 56-2A4) (Millipore MAB3309) were used. Whole slide images of the histology sections were acquired with an Axio-Scan microscope using Zen 2 core software at ×20 magnification and are presented without any subsequent processing. Digital image analysis was performed using Definiens AG (Munich) Tissue Studio 4.3 software. Tissue detection automatically identified all the tissue within each image, then a machine learning method was used to separate the sample from background and non-tissue regions, and segment the sample into dermis and epidermis; manual correction was used to ensure valid separation of these regions of interest (ROI). A fixed threshold was then applied to each ROI to identify the chromogen positive areas (μm^2^), which is represented as a percentage of the total tissue/ROI area.

## Results

### Derivation of TNF-Inducible Transcriptional Modules

Current anti-TNF therapies aim to block the bioactivity of TNF rather than expression or secretion of TNF. Therefore, measurement of TNF function rather than TNF levels is required to assess the effects of anti-TNF therapies. TNF exerts its biological functions *via* stimulation of the TNFRs and intracellular signaling cascades that converge on regulation of transcription factors and consequently gene expression (46). Our first aim was to derive and validate gene expression modules, which could be used to detect and quantify functional TNF activity. In MDM, we had previously reported one such module that discriminated cellular responses to stimulation with recombinant TNF (TNF-MDM module) from a selection of other cytokines including IFNγ, IL4, and IL13 (38). In the present study we reasoned that the transcriptional response to TNF stimulation may be influenced by its cellular context or the presence of other inflammatory mediators. Any individual gene expression module may be inadequate to detect all TNF activity. Therefore, we derived three novel TNF-inducible gene expression modules by alternative approaches.

**Figure 1:**
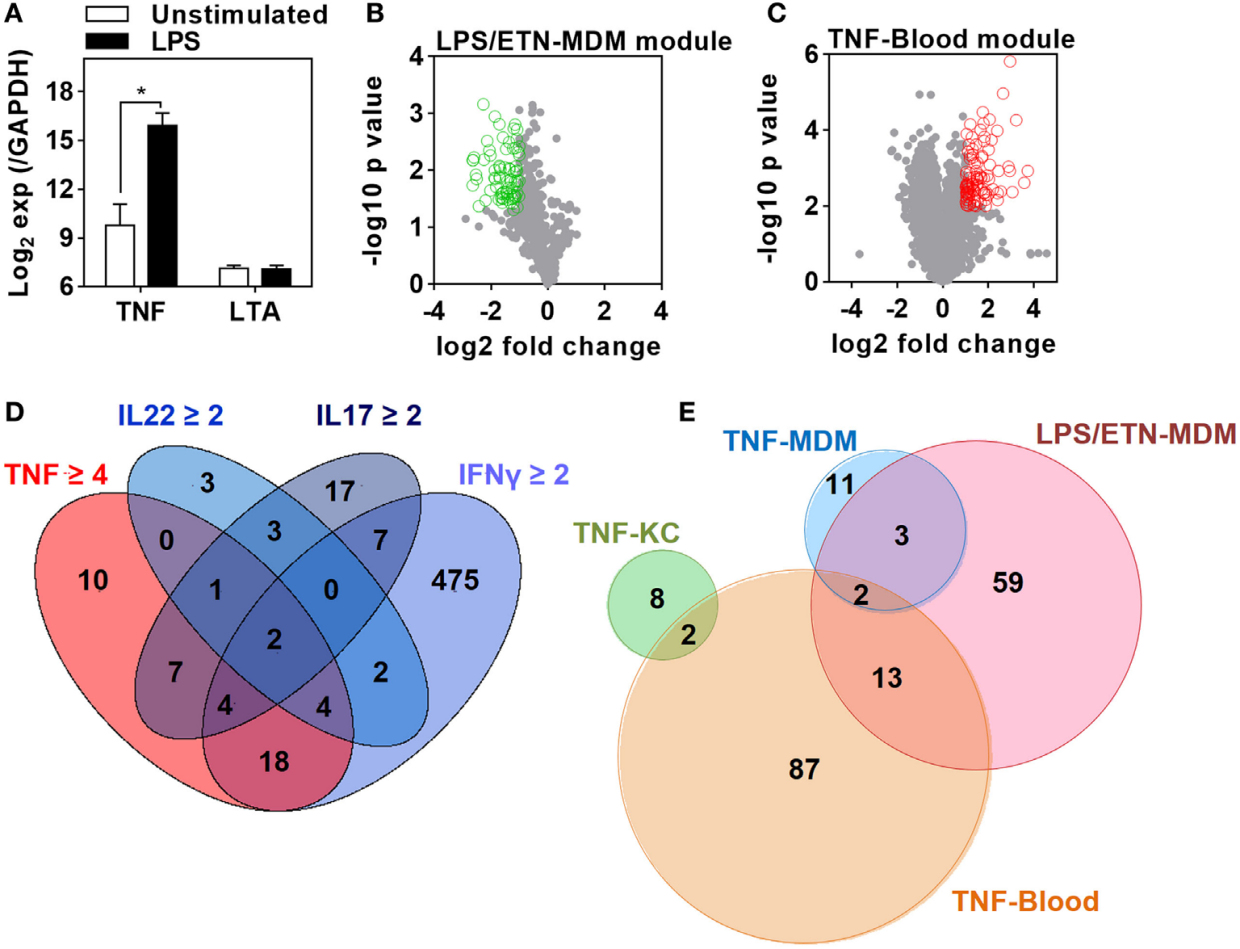
Derivation of transcriptional modules to measure tumor necrosis factor (TNF) activity. **(A)** Relative expression of TNF and lymphotoxin α (LTA) in monocyte-derived macrophage (MDM) cultures before and after 3 h stimulation with lipopolysaccharide (LPS) 100 ng/mL derived from previously published microarray data (ArrayExpress accession No: E-MEXP-1904 and E-MEXP-2032). Bars represent mean + SD (*n* = 3–5) and *p < 0.05, *t*-test. **(B)** Volcano plot of the effect of etanercept (ETN) on LPS-induced genes in MDM after 24 h. Open circles (green) represent 77 genes that were significantly (*p* < 0.05, *t*-test) attenuated (>2-fold) in the presence of ETN to give the “LPS/ETN-MDM” module. **(C)** Transcriptional response to TNF (10 ng/mL for 24 h) in *ex vivo* whole blood stimulation experiments. Open circles (red) represent 104 genes that were significantly (*p* < 0.01, *t*-test) upregulated (>2-fold) in response to TNF to give the “TNF-blood” module. **(D)** 4-way Venn diagram showing the overlap of keratinocyte (KC) genes significantly (*p* < 0.05, *t*-test) upregulated (>2-fold by IFNγ, IL22, IL17, or 4-fold by TNF) after 24 h stimulation. The 10 TNF-specific genes were used to give the “TNF-KC” module. **(E)** Venn diagram of the genes making up each TNF transcriptional modules, derived as described in panels **(B–D)** and by Bell et al. (“TNF-MDM”).

We first sought to identify gene expression attributable to TNF in the presence of other bioactive cytokines. We used innate immune stimulation of MDM with bacterial LPS as a prototypic stimulus to invoke secretion of a wide range of immunologically active factors, including TNF, contributing to LPS-inducible gene expression by their downstream autocrine and paracrine activity. To identify gene expression attributable to LPS-induced TNF, we compared the transcriptome of MDM 24 h after LPS stimulation in the presence and absence of ETN. Since ETN blocks the activity of LTA as well as that of TNF, we analyzed previously published data obtained from LPS stimulation in the same MDM model for induction of TNF and LTA expression (47, 48). As expected LPS induced robust upregulation of TNF expression in the primary transcriptional response (Figure 1A). This response would be expected to drive TNF-dependent gene expression changes at subsequent time points. In contrast, there was no induction of LTA. Therefore, gene expression attenuated by ETN in LPS-stimulated MDM unequivocally reflected endogenous TNF activity. We found significantly lower expression of 77 genes in the presence of ETN (Figure 1B), which we infer to be TNF responsive. We refer to these genes as the LPS/ETN-MDM module. In addition, we tested the effect of anti-TNF therapies on functional TNF activity in blood and derived a TNF gene expression module by identifying significantly upregulated genes following *ex vivo* whole blood stimulation with TNF (Figure 1C). We refer to this as the TNF-blood module. Our ultimate aim was to evaluate TNF activity in response to an *in vivo* immunological challenge using the TST (37, 38). Differential transcriptional responses to the same stimulus in different cell types are widely recognized (49). Therefore, we took advantage of previously published transcriptomic data from KCs stimulated with a selection of cytokines including TNF (44) to derive a TNF-specific module in keratinocytes (TNF-KC module) (Figure 1D).

**Figure 2:**
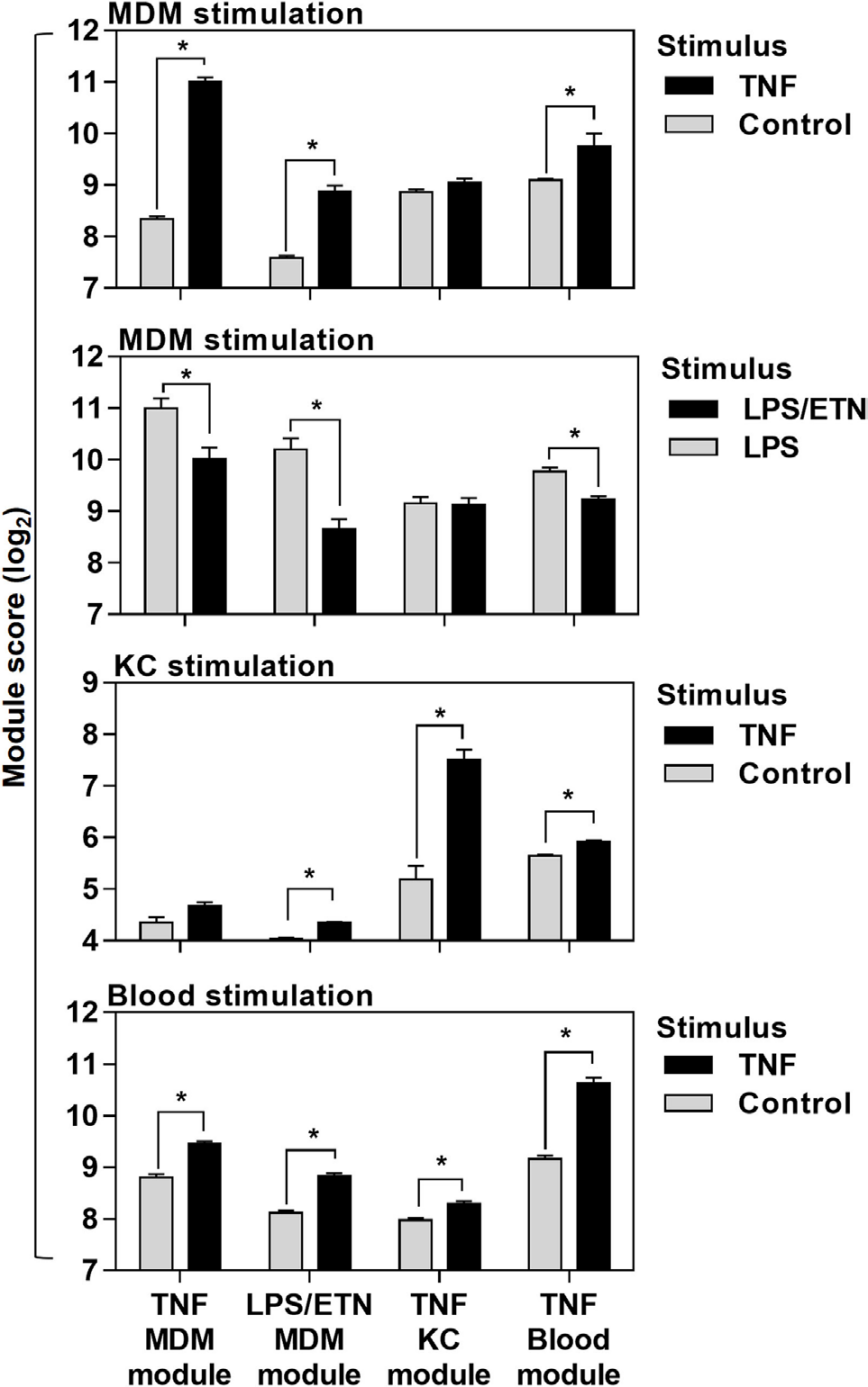
Cross-validation of transcriptional tumor necrosis factor (TNF) modules in the different experiments they were derived from. Module scores, calculated as geometric mean expression of the genes contributing to each module, in genome-wide transcriptomic data from monocyte-derived macrophages (MDM), keratinocytes (KC), and blood stimulated with ±TNF or lipopolysaccharide (LPS)-stimulated MDM ± etanercept (ETN). Bars represent mean + SD (*n* = 3–5). *p < 0.05, *t*-test.

Direct comparison of the genes included in each of the four modules (Data Sheet S1 in Supplementary Material) associated with TNF activity showed only modest overlap (Figure 1E). Cross-validation of each gene list in the different experiments which were used to derive them also highlighted their context specificity. Modules derived in MDM were not enriched in TNF-stimulated KCs and modules derived in KCs were not enriched in TNF- or LPS-stimulated MDM (Figure 2). Importantly, however, independent bioinformatic analysis of predicted upstream regulators of each of these gene lists showed statistically robust associations with TNF (Data Sheet S2 in Supplementary Material).

### Anti-TNF Therapy Attenuates TNF-Inducible Gene Expression in Blood

**Figure 3:**
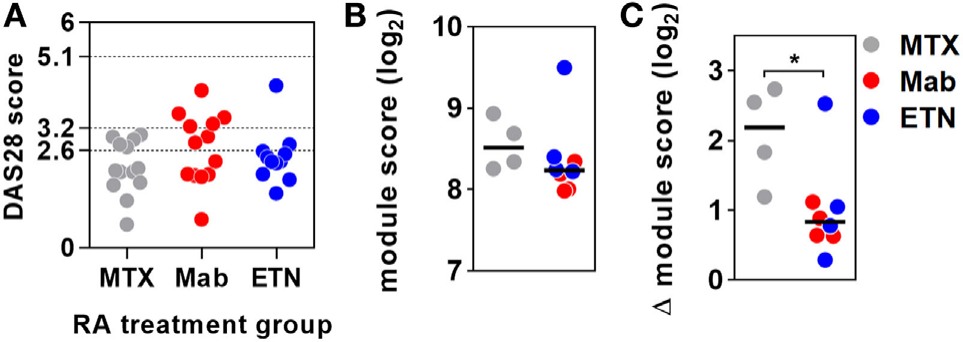
Anti-tumor necrosis factor (TNF) therapy attenuates TNF-inducible gene expression in blood. **(A)** Distribution of DAS28 scores in rheumatoid arthritis (RA) patients treated with methotrexate (MTX) only, anti-TNF antibodies (Mab), or etanercept (ETN). Dotted lines represent threshold values for disease in remission (<2.6), mild disease activity (<3.2), and moderate disease activity (<5.1). Transcriptional TNF activity, represented by the blood-derived TNF gene expression module score, **(B)** in peripheral blood of RA patients treated with MTX (*n* = 4), monoclonal anti-TNF antibodies (*n* = 4), or ETN (*n* = 4) at baseline, and **(C)** the change (Δ) in module score associated with TNF stimulation (10 ng/mL for 3 h). Data points were derived from individual experiments. Line represents the median. *p < 0.05, Mann–Whitney test.

Previous reports have interpreted the reduction in circulating pro-inflammatory mediators following initiation of anti-TNF therapies in RA as evidence for inhibition of TNF activity (17–19). Therefore, we reasoned that anti-TNF therapy would be associated with steady state reduction of TNF-dependent gene expression in blood and would attenuate upregulation of these gene expression modules following *ex vivo* stimulation with TNF. We quantified expression of the blood-TNF module before and after *ex vivo* TNF stimulation of blood from RA patients treated with anti-TNF therapies and compared these to blood samples from RA patients on MTX only. To avoid the confounding of differential disease activity, we restricted recruitment to patients on a stable treatment regimen for at least three months and who showed low levels of background inflammation, using C-reactive protein and the RA disease activity score of 28 joints (DAS28) (Table 2; Data Sheet S3 in Supplementary Material). There were no significant differences in DAS28 scores between RA patients in the different treatment groups (Figure 3A), as a biomarker of disease activity (42). All had a reduction of DAS28 score of >1.2 from pretreatment levels, thereby allowing them to continue on their current treatment regimen. None had a residual DAS28 score >5.1, which would represent ongoing highly active disease. In addition, the proportions of anti-cyclic citrullinated peptide antibody as a prognostic biomarker of disease (50) were not significantly different between the groups (Table 2).

Baseline levels of TNF-dependent gene expression were not significantly different in blood from RA patients on anti-TNF therapies compared to that of patients on MTX (Figure 3B). *Ex vivo* whole blood stimulation with TNF upregulated the expression of each of the TNF-dependent modules in all groups of patients, but this increase was significantly attenuated in patients on anti-TNF therapies (Figure 3C). In addition, we found that TNF was among the top predicted upstream regulators of all genes significantly attenuated by anti-TNF therapy in TNF-stimulated blood (Data Sheet S2 in Supplementary Material). Therefore, we concluded that in keeping with their expected effect, anti-TNF therapies attenuated the functional response to circulating TNF, which we modeled by *ex vivo* stimulation of peripheral blood.

### Quantitation of TNF Activity in the TST

**Figure 4:**
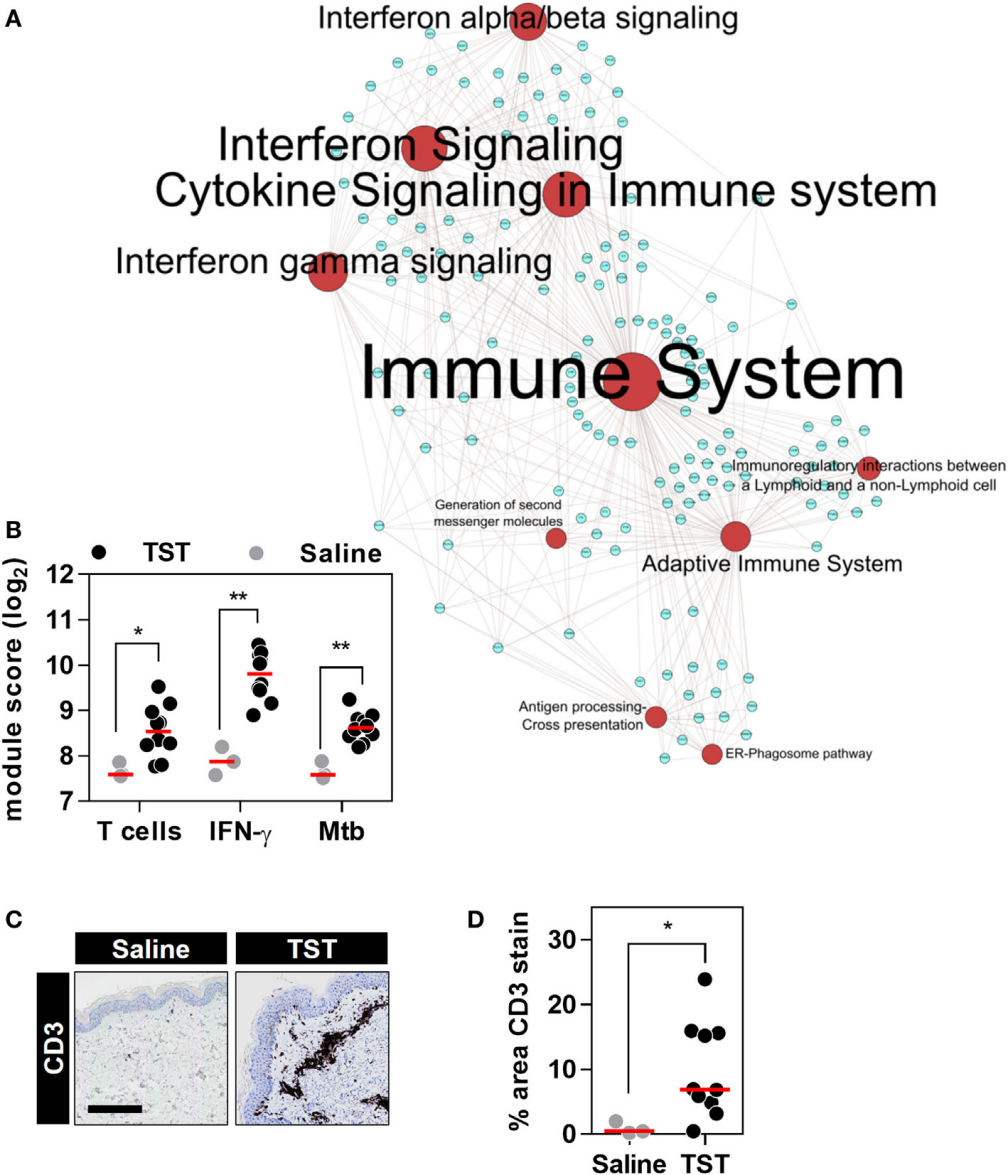
The transcriptional tuberculin skin test (TST) response of healthy volunteers. **(A)** Network graph of the top 10 Reactome pathways enriched among the genes upregulated in TST (*n* = 10) compared to saline (*n* = 3) samples from healthy volunteers. Red nodes represent pathways, and blue nodes represent genes. Node and label font size denote the significance of the respective pathway and are proportional to its −log_10_ *p* value. **(B)** Transcriptional module scores representing T cell recruitment, IFNγ activity, and responses to *Mycobacterium tuberculosis* (Mtb) in each biopsy. **(C)** Representative immunostaining of CD3 in skin biopsy samples from the site of TST and saline injections (scale bar = 500 µM). **(D)** Quantitation of CD3 immunostaining in each biopsy. Data points represent individual participants, and group medians are indicated by red lines. *p < 0.05, **p < 0.01 in Mann–Whitney tests.

**Figure 5:**
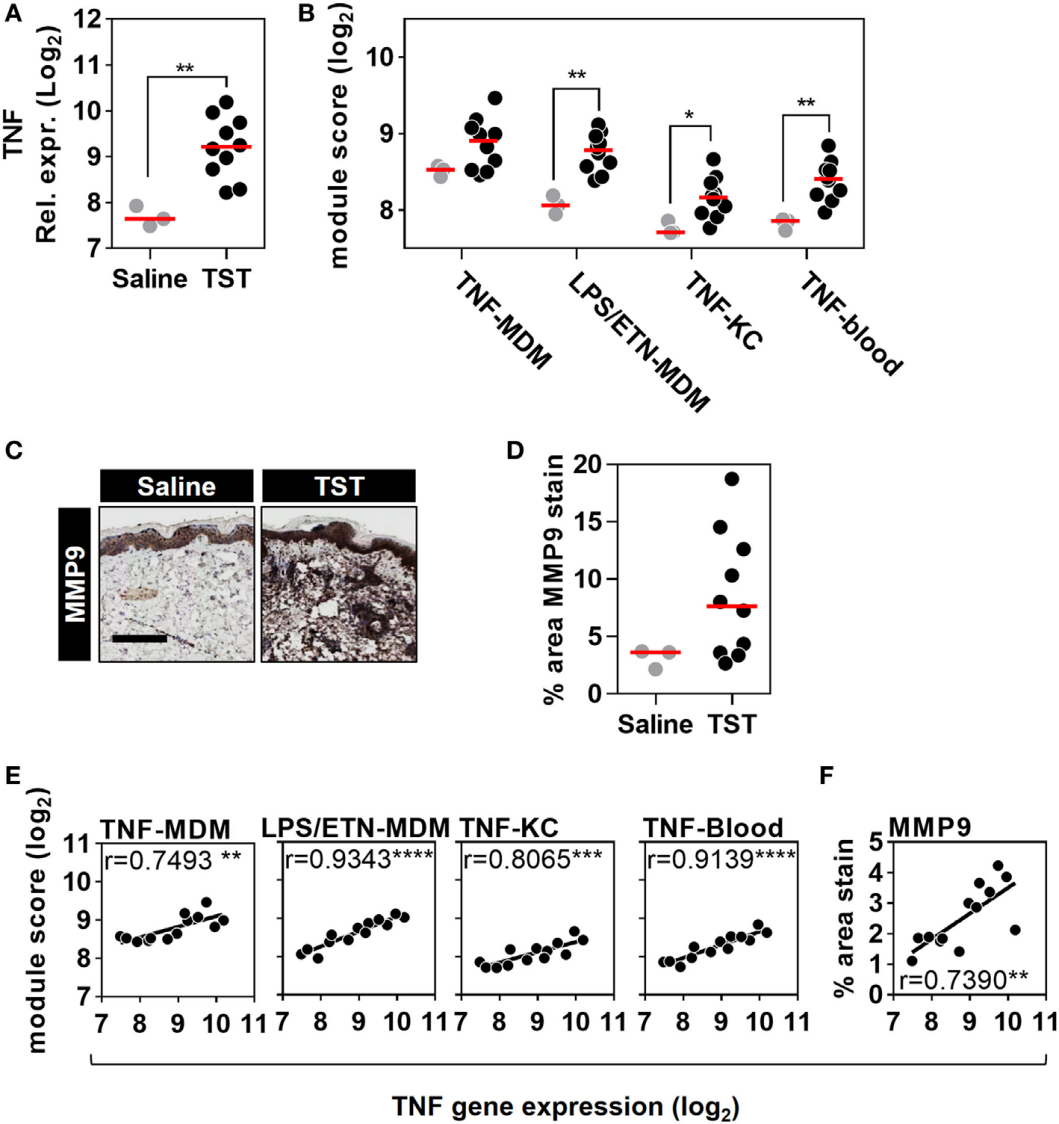
Quantitation of tumor necrosis factor (TNF) activity in the tuberculin skin test (TST) among healthy volunteers. **(A)** Relative TNF gene expression, **(B)** module scores of four separate TNF modules derived from genome-wide microarrays, and **(C)** representative immunostaining of matrix metalloproteinase (MMP) 9 in skin biopsy samples from the site of TST and saline injections (scale bar = 500 µM). **(D)** Positive immunostaining for matrix metalloproteinase (MMP) 9 in skin biopsies from the site of TST (*n* = 10) or control saline injections (*n* = 3) in healthy volunteers. Data points represent individual experiments. *p < 0.05, **p < 0.01, Mann–Whitney test. **(E,F)** Correlation between TNF expression and each of the TNF module scores or positive log_2_ MMP9 immunostaining. Correlation coefficients (*r*) and *p* values were derived from Pearson correlation analyses. **p < 0.01, ***p < 0.001, ****p < 0.0001.

Next, we tested the effect of anti-TNF therapies on TNF activity at the site of an immune challenge *in vivo*. To do so, we undertook transcriptional profiling of skin biopsies from the site of TSTs in individuals with antimycobacterial T cell memory (Table 2; Data Sheet S3 in Supplementary Material). In healthy volunteers, the TST invokes a multivariate immune response that comprises innate immune responses, immune cell recruitment, and cytokine responses (Figure 4; Data Sheet S4 in Supplementary Material) (37, 38). This includes robust enrichment of TNF gene expression itself and of all four TNF-dependent gene expression modules derived from MDM, KCs, and blood cells, representing a broad range of TNF-responsive cells in the context of the TST (Figures 5A,B).

To extend the assessment of TNF activity beyond transcriptional responses to the protein level, we also immunostained TST biopsy sites to quantify expression of the MMP9 (Figure 5C). MMP9 has been associated with TNF activity in previous studies (51). It is also a component of the TNF-KC module and therefore a specific protein target in the skin to discriminate between TNF and IFNγ activity (Figure 1D; Data Sheet S1 in Supplementary Material). Six of the ten healthy volunteers undergoing TST injection showed increased MMP9 protein expression compared to participants receiving control saline injections (Figure 5D). The variability in TNF modules and MMP9 immunostaining each demonstrated a statistically significant correlation with TNF transcript levels among different individuals (Figures 5E,F). These correlations consolidated the evidence for a functional relationship between TNF expression and the independently derived TNF-inducible transcriptional modules or MMP9 immunostaining. Hence, we conclude that the TST invokes a functional TNF response, within which we can quantify variation in TNF bioactivity.

### Functional TNF Activity Is Preserved at the Site of an Immunological Challenge *In Vivo* Despite Anti-TNF Therapy

**Figure 6:**
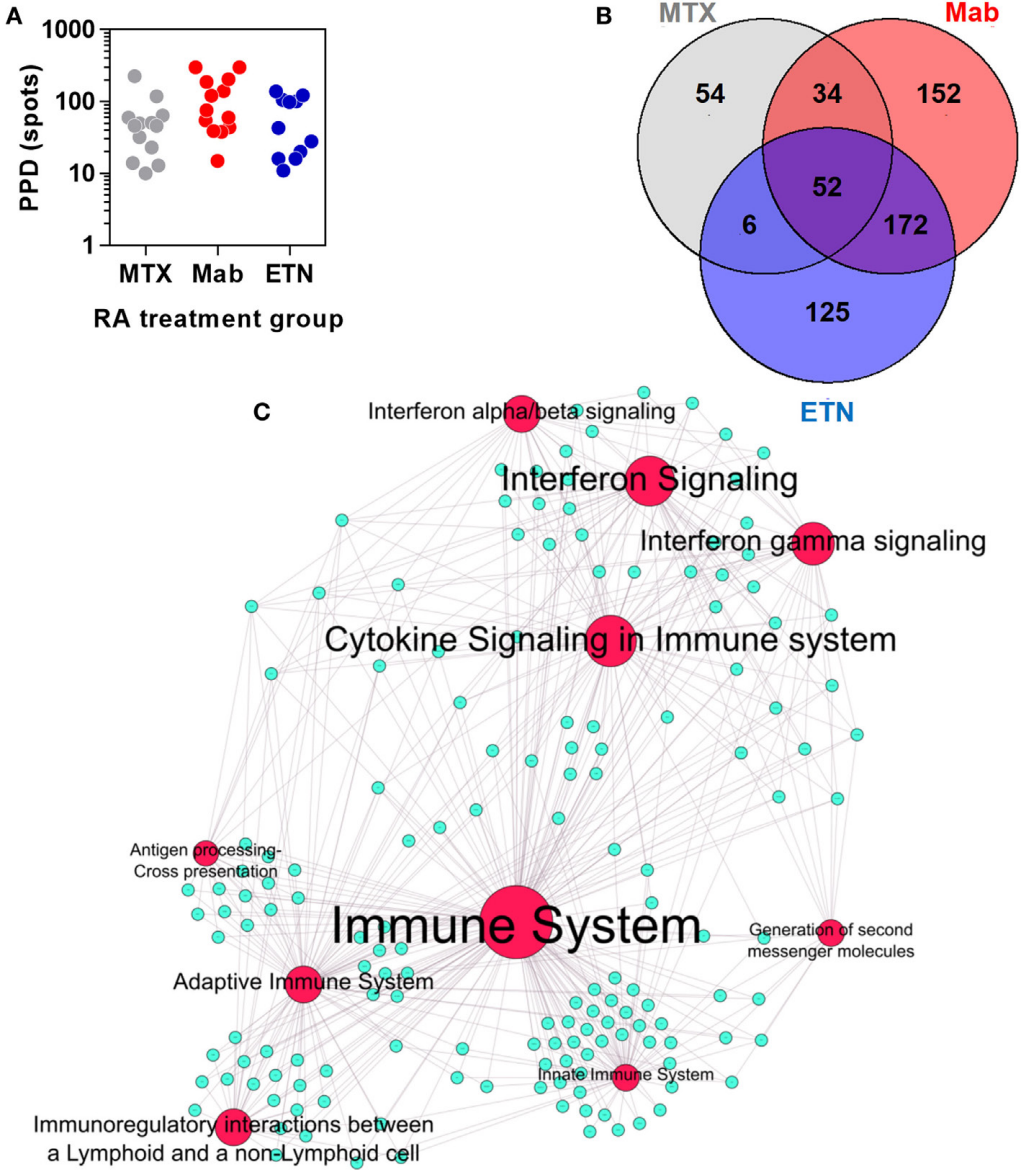
The transcriptional tuberculin skin test (TST) response of rheumatoid arthritis (RA) patients. **(A)** Distribution of quantitative IFNγ ELISpot results in RA patients treated with methotrexate (MTX) only, anti-TNF antibodies (Mab), or etanercept (ETN). Each data point represents an individual patient. **(B)** Number and overlap of genes upregulated in TST compared to saline samples in patients treated with MTX, monoclonal anti-TNF antibodies (Mab), or the soluble tumor necrosis factor (TNF) receptor ETN. The total number of genes displayed in this Venn diagram was combined in the integrated TST module. **(C)** Network graph of the top 10 Reactome pathways enriched in the integrated TST signature genes. Red nodes represent pathways, and blue nodes represent genes. Node and label font size denote the significance of the respective pathway and are proportional to its −log_10_ *p* value.

Rheumatoid arthritis patients treated with anti-TNF therapies or MTX only, had comparable peripheral blood IFNγ ELISpot responses to tuberculin/PPD stimulation (Figure 6A), indicating that these study groups had equivalent T cell memory responses for tuberculin irrespective of any differences in their past history of TB exposure or treatment. In these participants, we defined an integrated TST transcriptional signature. This signature comprised 595 genes that were significantly enriched in the TST compared to saline injection sites in at least one study group (Figure 6B). Bioinformatic analysis of these genes reflected the same immune pathways evident in the TST transcriptome of healthy individuals (Figure 6C).

**Figure 7:**
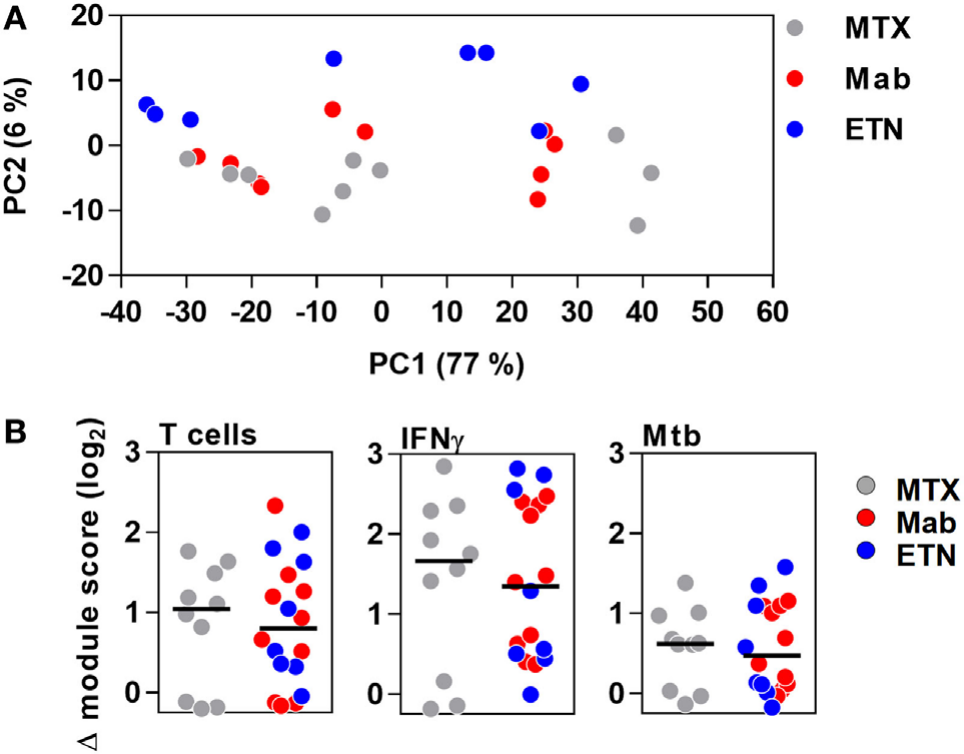
The transcriptional tuberculin skin test (TST) response in patients with rheumatoid arthritis. **(A)** Principal component (PC) analysis of integrated TST signature genes showed no treatment-specific clustering in PC1 and PC2, which together explained 83% of variation in this data set. Data points represent individual experiments. **(B)** Transcriptional modules representing enrichment of T cells, IFNγ activity, and innate immune responses to *Mycobacterium tuberculosis* (Mtb) in TST compared to saline baseline as shown by log_2_ module enrichment scores. Individual patients are represented with separate data points, and group medians are indicated by black lines. MTX, methotrexate; Mab, monoclonal anti-TNF antibodies; ETN, etanercept.

Principal component analysis of the integrated TST signature genes, as well as enrichment of T cell-associated and IFNγ-dependent gene expression modules, indicated that the major features of transcriptional responses in the TST of RA patients were not affected by anti-TNF therapy (Figure 7).

**Figure 8:**
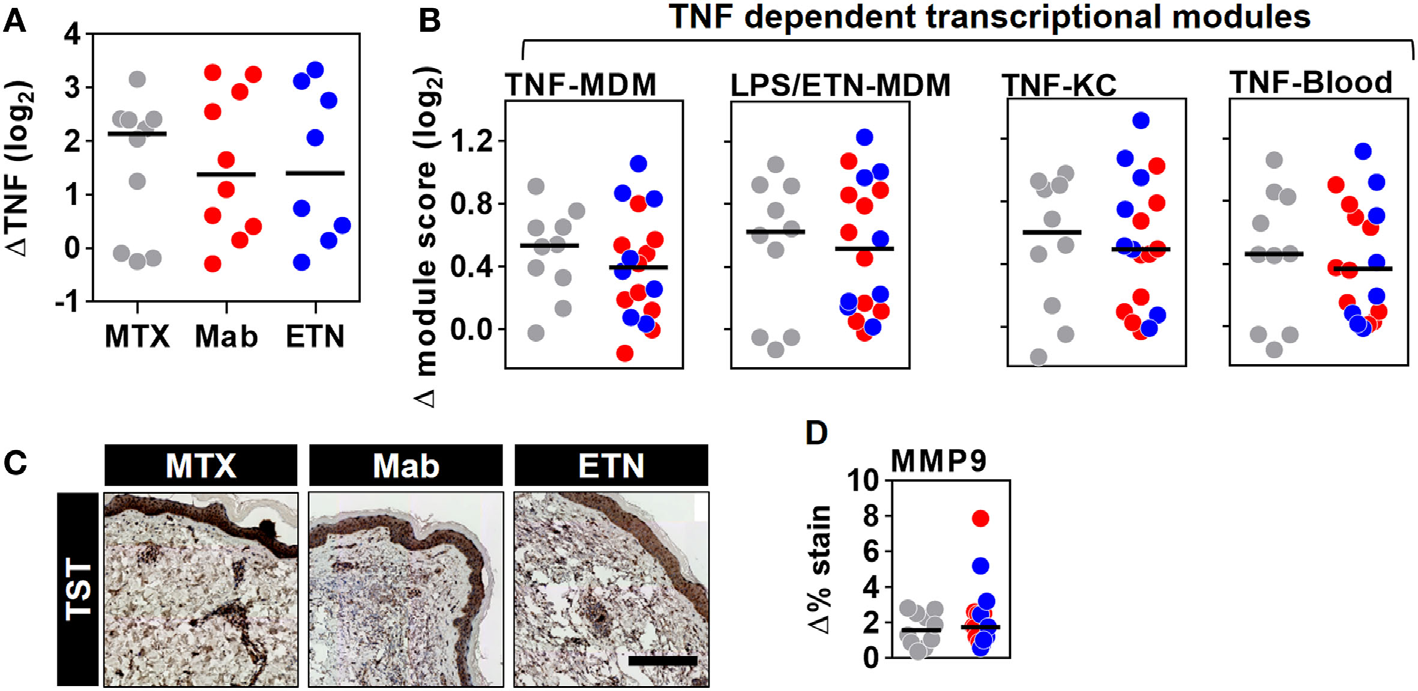
Inducible tumor necrosis factor (TNF) activity is preserved in the TST of rheumatoid arthritis (RA) patients despite anti-TNF therapy. The change in **(A)** TNF gene expression and **(B)** each of the four TNF-dependent gene expression modules in TST skin biopsies from RA patients treated with methotrexate (MTX, *n* = 10), monoclonal anti-TNF antibodies (Mab, *n* = 10), or etanercept (ETN, *n* = 8), compared to control saline injections (*n* = 3 per group). **(C)** Representative images of MMP9 immunohistochemical staining (scale bar = 500 μm) and **(D)** positive MMP9 staining in skin biopsies from the same patients. Data points represent individual experiments. Line represents the median. TST, tuberculin skin test; MDM, monocyte-derived macrophage; LPS, lipopolysaccharide; KC, keratinocyte; MMP9, matrix metalloproteinase 9.

The TST response in RA patients included enrichment of TNF gene expression, which was similar in all patient groups and therefore not affected by anti-TNF therapy (Figure 8A). Surprisingly, however, comparable levels of the TNF-dependent gene expression modules were also observed in all the study groups (Figure 8B). Likewise, MMP9 immunostaining representing a protein biomarker of TNF activity was comparable in anti-TNF treated and control patients (Figures 8C,D). Therefore, we conclude that anti-TNF therapy in RA patients does not inhibit inducible TNF function during a prototypic cell-mediated immune response at the site of immune challenge.

## Discussion

The rationale for the development of anti-TNF therapies in RA was based on local expression of TNF at the site of disease, our understanding of its functional role in pro-inflammatory immune cell recruitment to tissues and data from animal models in which over expression of human TNF caused a chronic inflammatory arthritis that could be ameliorated by anti-TNF therapy (52). In this context, the unequivocal therapeutic effects of anti-TNF antibodies and ETN in RA led to two conclusions. First, anti-TNF therapies mediate their beneficial effect by inhibiting TNF activity at the site of disease and consequently, TNF function makes an essential contribution to the immunopathogenesis of disease. Second, the same rationale applied to the detrimental effect of TNF inhibition, as increased risk of certain infections among patients on anti-TNF therapies (31, 53) suggested that TNF activity is an essential component of protective immune responses.

Several lines of evidence suggest that the effects of anti-TNF agents are not wholly understood. For example, it has become evident that anti-TNF antibodies and ETN have differential biological activity. Although they mediate similar therapeutic effects in RA, anti-TNF antibodies but not ETN are effective in other chronic inflammatory disease such as Crohn’s colitis (54). Likewise, the use of anti-TNF antibodies incurs significantly greater risk of active tuberculosis than that of ETN (30–32). In addition, we have previously shown that anti-TNF antibodies, but not ETN, induce increased emergence of functionally active regulatory T cells (20). Counterintuitively, this last example may be mediated by augmentation of TNF function through enhancing the interaction of membrane-bound TNF and TNFR2 (21). Therefore, the biological effects of anti-TNF therapies may extend beyond the canonical view that they block TNF activity at the site of immune responses in tissue. In fact, there are no experimental data that conclusively show this effect in man.

To test this question directly, we adopted a human experimental challenge approach using the TST as a standardized stimulus with which to invoke a focus of cell-mediated immune responses (37). Transcriptional profiling from skin punch biopsies from the site of the TST afforded us the opportunity to make comprehensive genome-wide assessments of immune responses at the molecular level with unprecedented sensitivity. We complemented this approach with the generation of independent experimentally derived TNF-dependent gene expression modules representing the functional bioactivity of TNF. Our evaluation of TNF activity at the transcriptional level revealed striking differences between TNF-dependent transcriptional responses in macrophages, blood cells, and KCs. The mechanisms underlying the context specificity in the functional activity of TNF likely reflect distinct epigenetic landscapes in different cell types (55). Despite the limited overlap between each of the four TNF-dependent gene expression modules we derived, *in silico* bioinformatics analysis yielded TNF as one of the top predicted upstream transactivator of each list of genes. These independently derived transcriptional modules associated with different cell types present at the site of the TST, namely, KCs, macrophages, and recruited blood leukocytes enhanced the sensitivity of our analysis and provided cross-validation.

In healthy subjects, we showed that TNF expression was enriched in the TST and that the variability in its enrichment correlated with concordant enrichment of each of the four TNF-dependent transcriptional modules. In addition, we extended this analysis to the protein level, by showing correlation with MMP9 as a TNF-inducible protein (56). Taken together, these data showed unequivocally that the TST invokes a functional TNF response and that we can quantify variability in TNF bioactivity *in situ* within this response.

To test the effects of anti-TNF agents, we sought to compare RA patients with quiescent disease on MTX to those receiving anti-TNF agents. In blood, baseline TNF bioactivity represented by the expression of TNF modules was not significantly different between RA patients on MTX and those on anti-TNF therapies. Therefore, our data suggest that the widely reported diminution of pro-inflammatory mediators in RA patients with quiescent disease following anti-TNF therapy may not be related to a targeted reduction in steady state expression of TNF-dependent gene expression but could simply represent successful control of active disease in patients on treatment with MTX or anti-TNF therapies, albeit through possibly divergent mechanisms. Nonetheless, we confirmed that enrichment of TNF-dependent gene expression following *ex vivo* TNF stimulation of whole blood was significantly attenuated in blood from patients on anti-TNF agents. These findings are in keeping with previous reports of neutralization of TNF bioactivity by plasma from patients receiving infliximab (57, 58). However, in the same groups of patients, receipt of anti-TNF agents did not attenuate the enrichment of the TNF-dependent transcriptional modules or MMP9 immunostaining within the TST. Hence, we concluded that although anti-TNF agents can inhibit TNF bioactivity in the circulation, they have no discernible effect on *in vivo* TNF activity at the site of an acute immune challenge.

Our data fundamentally challenge the long held view that anti-TNF agents mediate their therapeutic effect in RA by targeting TNF activity at the site of disease. We speculate that anti-TNF agents do not reach sufficient concentration within the tissue microenvironment to neutralize the level of TNF produced in an acute cell-mediated immune response. It remains possible that they can inhibit lower levels of TNF at foci of chronic inflammation. Alternatively, their therapeutic effects may be mediated by neutralizing the actions of circulating TNF, suggesting a previously unrecognized model by which TNF may contribute to the pathogenesis of RA. Further evaluation of this hypothesis may yield new mechanistic insights into the systemic immunopathology of RA and other inflammatory diseases amenable to anti-TNF therapies.

## Ethics Statement

This study was approved by UK National Research Ethics Service (reference no: 11/LO/1863), and written informed consent was obtained from all participants.

## Author Contributions

RB-M, MRE, and MN designed the experiments. RB-M, CT, NG, and LB conducted the experiments. RB-M, CT, GP, ME, and MN undertook the analysis. RB-M, CT, MRE, and MN wrote the paper with input from all other authors.

## Conflict of Interest Statement

The authors declare that the research was conducted in the absence of any commercial or financial relationships that could be construed as a potential conflict of interest.

## Footnotes

^1^https://www.ebi.ac.uk/arrayexpress/.

^2^http://mev.tm4.org/.

^3^http://www.innatedb.com.

^4^https://gephi.org/.

^5^www.r-project.org.
